# Relentless Placoid Chorioretinitis: A Differential Diagnosis and Management Approach in a Challenging Case

**DOI:** 10.7759/cureus.88688

**Published:** 2025-07-24

**Authors:** Jan Bombuy Gimenez, Monika Lazicka-Galecka, Maria Guszkowska, Jacek P Szaflik

**Affiliations:** 1 Department of Ophthalmology, Medical University of Warsaw, Warsaw, POL

**Keywords:** apmppe, covid-19, immunomodulatory therapy, inflammatory chorioretinopathies, relentless placoid chorioretinitis, serpiginous choroiditis, white dot syndrome

## Abstract

This case study presents a 51-year-old male diagnosed with relentless placoid chorioretinitis (RPC), a rare condition within the spectrum of inflammatory chorioretinopathies, who experienced progressive vision loss following a COVID-19 infection. The patient's clinical presentation was accompanied by a similar episode, misdiagnosed and untreated, nine years earlier.

During clinical evaluation, the patient exhibited overlapping features of acute posterior multifocal placoid pigment epitheliopathy (APMPPE), serpiginous choroiditis (SC), and RPC. Fundoscopic examination revealed bilateral chorioretinal involvement, including characteristic placoid lesions in the posterior pole resembling APMPPE, as well as serpentine, amoeboid-shaped peripheral lesions similar to SC. Optical coherence tomography (OCT), autofluorescence imaging, and angio-OCT demonstrated active inflammatory lesions, along with chronic atrophic changes, reflecting different stages of the disease. The article further discusses the differential diagnosis, considering inflammatory chorioretinopathies, systemic disease-related chorioretinopathies, and other conditions with a similar clinical course.

This case study presents the recommended therapeutic strategy for RPC: triple immunomodulatory therapy (IMT) consisting of cyclosporine A, mycophenolate mofetil, and methylprednisolone, which resulted in clinical remission six months after therapy initiation in the reported patient. The article highlights the necessity of recognizing the chronic and progressive nature of RPC, as well as the need for aggressive immunosuppressive treatment rather than corticosteroid monotherapy.

## Introduction

Inflammatory chorioretinopathies represent a group of ocular inflammatory conditions that pose significant diagnostic and therapeutic challenges for ophthalmologists. These conditions, frequently misdiagnosed, may progress if inadequately treated, leading to marked retinal inflammation, secondary atrophy, and ultimately irreversible vision loss or, in severe cases, complete blindness [1,2]. This article presents the case of a patient who was referred to our clinic from another hospital more than two months after the onset of symptoms during the current disease flare. Previously, the patient had experienced a similar episode in one eye nine years earlier, which had remained improperly diagnosed and was not adequately treated.

The purpose of this case study is to highlight the clinical characteristics of relentless placoid chorioretinitis (RPC), with a focus on its disease profile, imaging-based diagnostics, and differential diagnosis with other conditions within the spectrum of inflammatory chorioretinopathies (formerly known as white dot syndromes).

## Case presentation

A 51-year-old Caucasian male was referred to the Ophthalmology Clinic from his hometown ophthalmology hospital due to significant vision loss. The gradual decline in vision began two months prior to his first visit to the clinic, following a symptomatic COVID-19 infection. In the meantime, the patient underwent ophthalmologic follow-up at the referring hospital, where he received glucocorticosteroid treatment for over a month, without clinical improvement. The patient's ophthalmic history revealed that he had experienced visual flickering in the left eye nine years earlier. The episode followed a similar course to the current presentation. At that time, extensive retinal and choroidal atrophy was diagnosed in the left eye, although no definitive underlying cause was established. Since then, the patient had not undergone any further ophthalmologic evaluation. The patient reports that the right eye remained unchanged; however, this year’s optical coherence tomography (OCT) examination revealed progression of retinal degeneration compared to the exam conducted nine years ago, at which time the eye had not been diagnosed with any abnormalities.

**Figure 1:**
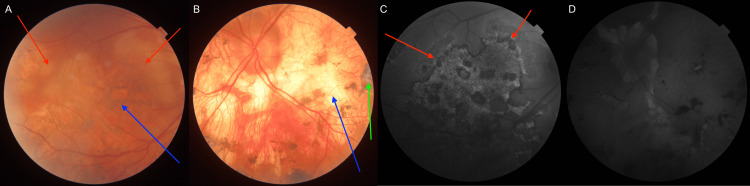
Fundoscopic and fundus autofluorescence images demonstrating ocular pathological changes on the day of admission to the clinic (A) Fundoscopic image of the right eye (red arrows indicate active lesions, while the blue arrow indicates old inactive changes). (B) Fundoscopic image of the left eye (the blue arrow indicates old atrophic changes, while the green arrow indicates subretinal fibrosis). (C) Fundus autofluorescence image of the right eye, showing hyperautofluorescent signals (marked with red arrows) corresponding to active inflammatory lesions. (D) Fundus autofluorescence image of the left eye, showing hypoautofluorescent areas corresponding to old atrophic changes.

On physical examination, the corrected distance visual acuity was VOD 0.6 sc and VOS 0.2 sc. Near vision was Snellen OD 0.5 cc and OS 2.4 cc (single letters). Intraocular pressure, measured by Goldmann tonometry, was 9 mmHg in the right eye and 15 mmHg in the left eye. Color vision was impaired in the left eye. The anterior segment of both eyes was without abnormalities. In the vitreous body of the right eye, fine granular lesions were observed in the anterior portion; the left eye was clear and transparent. Fundoscopic examination demonstrated a heterogeneous pattern of chorioretinal involvement. Numerous round, placoid lesions were observed within the posterior pole, consistent with features typically described in acute posterior multifocal placoid pigment epitheliopathy (APMPPE). In addition, the peripheral retina exhibited extensive serpentine, amoeboid-shaped lesions, similar to those found in the course of serpiginous choroiditis (SC). Dispersed choroidal foci that extended into the deeper retinal layers further complicated the clinical picture. In the right eye, both active inflammatory lesions and atrophic changes with pigment migration were present, reflecting different stages of disease evolution. The yellowish lesions had irregular borders, and although the macular region was involved, it remained free of edema - an observation that nonetheless raised concerns regarding the potential diagnosis and prognosis (Figures 1A, 1C). The lesions did not extend beyond the equator. In the left eye, the optic disc appeared pale and atrophic, with massive peripapillary and posterior pole retinal atrophy, partially pigmented (old changes) (Figures 1B, 1D).

**Figure 2:**
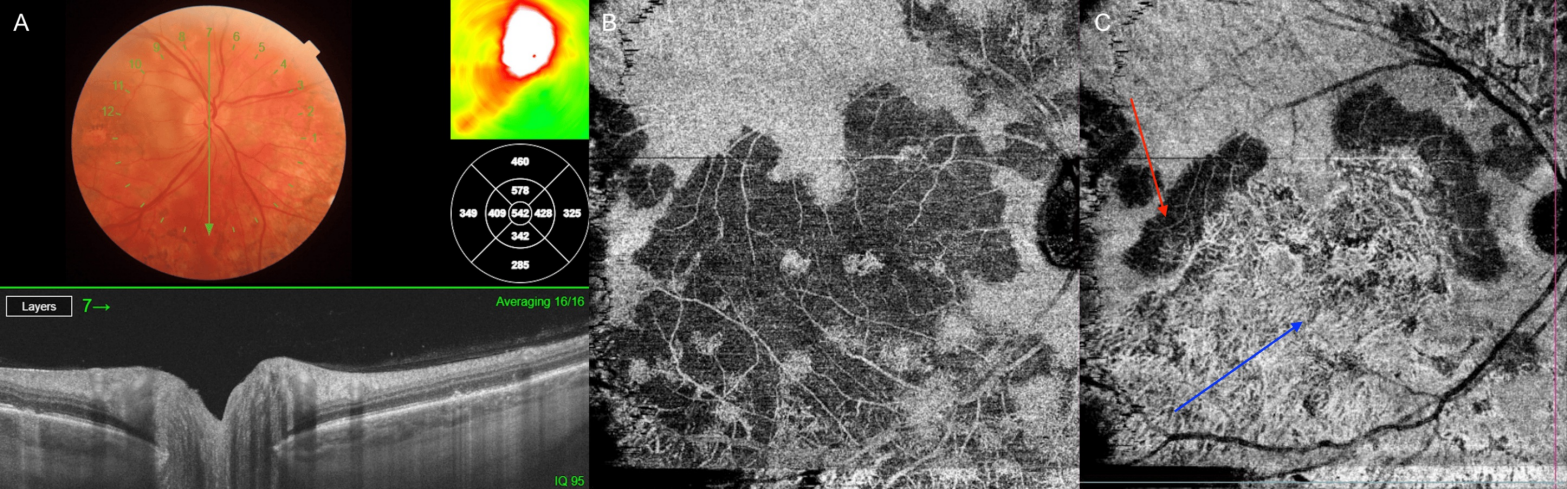
OCT and angio-OCT of the right eye on the day of admission (A) OCT image showing optic disc edema in the right eye. (B) Angio-OCT image of the choriocapillaris layer, showing hypoperfusion of the choriocapillaris. (C) Angio-OCT image of the choroidal layer, showing choroidal atrophy and active inflammatory lesions (the red arrow indicates active hyporeflective changes, while the blue arrow indicates old hyperreflective changes). OCT, Optical coherence tomography

**Figure 3:**
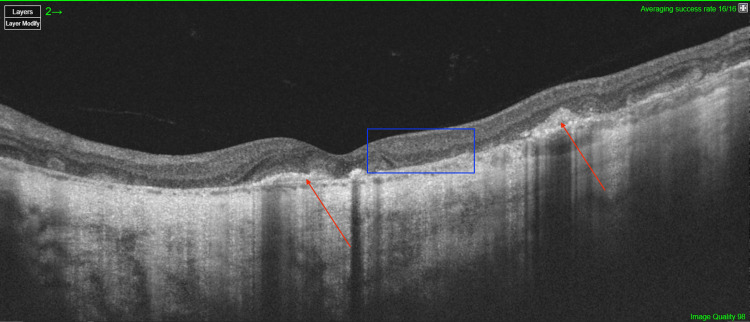
OCT of the left eye on the day of admission OCT image showing the absence of photoreceptor and choroidal layers, as well as full-thickness retinal thinning. Red arrows indicate subretinal fibrosis, while the blue square marks disrupted retinal layer architecture. OCT, Optical coherence tomography

OCT of the right eye confirmed optic disc edema and extensive outer retinal layer loss in inactive lesions, accompanied by choroidal thinning (Figure 2A). In active areas, choroidal edema was present, with disruption of the inner choroidal layers and associated edema of the retinal nerve fiber layer (RNFL), with a visible lesion. Angio-OCT of the right eye revealed extensive choroidal alterations (Figures 2B, 2C). Autofluorescence imaging showed hyperautofluorescent signals corresponding to active lesions and hypoautofluorescent areas representing chronic atrophic changes (Figure 1C). In the left eye, OCT demonstrated significant choroidal and full-thickness retinal atrophy (Figure 3).

The clinical presentation exhibited features overlapping with three closely related entities: RPC, APMPPE, and SC. These diagnoses represented an indication for initiating triple immunomodulatory therapy (IMT), which included the administration of the following medications: cyclosporine A 2 × 50 mg for seven days, followed by dose doubling; mycophenolate mofetil 2 × 1000 mg; methylprednisolone starting at 1 × 40 mg with progressive dose reduction; and omeprazole 1 × 20 mg. Furthermore, necessary supplementation with vitamin D3 (min 2000 IU/day) and calcium (min 800 IU/day) was recommended. As part of the pre-treatment preparation, a laboratory investigation was performed. A positive Quantiferon test was detected, which later proved to be a false-positive result. The patient was consulted by a pulmonologist who, based on chest X-ray imaging, did not suspect tuberculosis. For certainty, biochemical culture studies were performed, which excluded potential disease. The laboratory findings revealed leukocytosis with marked granulocytosis and a lymphocyte count at the upper limit of normal, consistent with a hematologic response typically associated with systemic corticosteroid treatment (WBC: 17.09 × 10³/μL (ref. 4.23-9.07); neutrophils: 13.25 × 10³/μL (ref. 2.00-7.00); immature granulocytes (IG): 0.08 × 10³/μL (ref. 0.00-0.03); lymphocytes: 2.9 × 10³/μL (ref. 1.00-3.00)). A hematologic consultation was performed, which excluded any clinically significant underlying pathology.

After more than six months of IMT, disease progression was successfully suppressed, and the retinal inflammatory foci were brought into remission. Nevertheless, the patient experienced a decline in visual acuity, with distance best-corrected visual acuity measured at 0.08 in the right eye and 0.3 in the left eye, and near visual acuity in both eyes declining to 3.6 cc.

**Figure 4:**
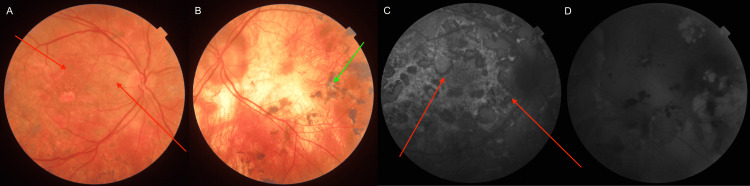
Comparison of fundoscopic and fundus autofluorescence imaging findings obtained following one month after the initiation of IMT (A) Fundoscopic image of the right eye (red arrows show regression of previous lesions). (B) Fundoscopic image of the left eye (the green arrow shows subretinal fibrosis). (C) Fundus autofluorescence image of the right eye (red arrows show the transition of previously hyperautofluorescent active lesions into hypofluorescent areas). (D) Fundus autofluorescence image of the left eye. IMT, Immunomodulatory therapy

**Figure 5:**
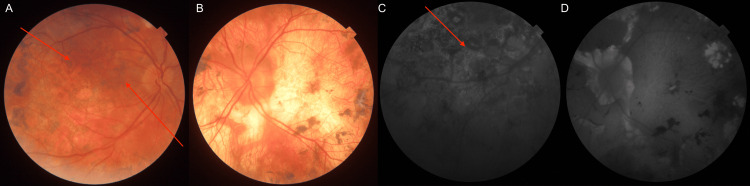
Comparison of fundoscopic and fundus autofluorescence imaging findings obtained following three months after the initiation of IMT (A) Fundoscopic image of the right eye (red arrows show further healing of previously active lesions). (B) Fundoscopic image of the left eye. (C) Fundus autofluorescence image of the right eye (the red arrow shows the healing process). (D) Fundus autofluorescence image of the left eye. IMT, Immunomodulatory therapy

**Figure 6:**
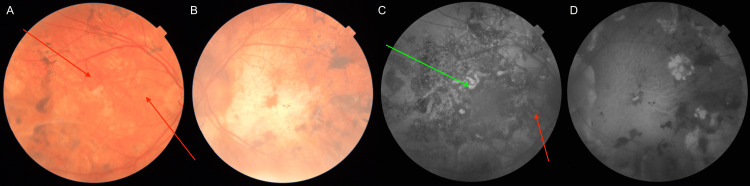
Comparison of fundoscopic and fundus autofluorescence imaging findings obtained following six months after the initiation of IMT (A) Fundoscopic image of the right eye (red arrows show inactivation of previous lesions). (B) Fundoscopic image of the left eye. Retinal atrophic changes are visible. (C) Fundus autofluorescence image of the right eye (the red arrow shows inactivation of lesions (hypofluorescent signal), while the green arrow shows subretinal fibrosis). (D) Fundus autofluorescence image of the left eye. The image shows hypofluorescent changes consistent with inactive lesions. IMT, Immunomodulatory therapy

The figures below present a comparison of fundoscopic and autofluorescence imaging findings obtained at one (Figure 4), three (Figure 5), and six months (Figure 6) following the initiation of triple IMT. The images demonstrate a gradual reduction in active inflammatory lesions and no evidence of disease progression over time. Lesion borders become less blurred, with pigment accumulation appearing; what’s more, the lesions evolve into inactive scars. By six months, the changes show no signs of activity, evidenced by the absence of hyperfluorescence at the lesion edges on autofluorescence imaging.

## Discussion

The characteristic appearance in diagnostic examinations indicated, from the beginning, that the patient was suffering from a disease potentially belonging to the former white dot syndrome group. This group encompasses inflammatory chorioretinopathies, whose common clinical feature is the presence of numerous well-demarcated white lesions located in the deeper layers of the retina and choroid, visible on fundoscopic examination as multifocal foci [1]. These diseases are typically preceded by a viral infection and most commonly occur in individuals between the second and sixth decades of life [3].

In the differential diagnosis, several inflammatory chorioretinopathies - such as multiple evanescent white dot syndrome (MEWDS), multifocal choroiditis with panuveitis (MCP), birdshot chorioretinopathy (BCR), and punctate inner choroidopathy (PIC) - could be immediately excluded. MEWDS, in contrast to the disease affecting the described patient, is typically unilateral and self-limiting, with lesions resolving within weeks to months. The clinical picture of MCP is characterized by anterior uveitis, significant vitreous inflammation, and characteristic punched-out chorioretinal scars - all of which are absent in the presented case. BCR, on the other hand, differs in diagnostic imaging, as it typically presents with creamy, oval-shaped lesions clustered around the optic disc, accompanied by significant vitreous inflammation and retinal vasculitis. Furthermore, the presence of the HLA-A29 antigen is a key differentiating factor. PIC typically affects young, myopic women and presents with small, limited lesions localized to the posterior pole [3].

Of the diseases classified under the inflammatory chorioretinopathies, only three corresponded directly to the clinical presentation observed in the patient: APMPPE, SC, and RPC. APMPPE could be considered a potential diagnosis, as bilateral placoid lesions at the level of the retinal pigment epithelium are present in the case described. However, APMPPE typically manifests as an acute, self-limited condition that resolves completely in a matter of weeks to months [4,5]. This contrasts with the patient’s progressive course, continuous active inflammation, and positive response to immunosuppressive treatment. SC represents a strong differential consideration, given the characteristic serpentine pattern of choroidal inflammation extending centrifugally from the peripapillary region and its chronic, progressive nature, with choroidal thickening and outer retinal disruption. SC typically presents with serpentine choroidal lesions that spread from the optic disc, with minimal vitritis [6]. However, this case exhibits atypical features, such as bilateral involvement in different stages of the disease and dense vitreous inflammation, which could indicate a more aggressive variant of SC or an alternative diagnosis like RPC, which is regarded as a variant within the SC spectrum. The chronicity, the combination of bilateral placoid lesions in various stages of evolution, the relentless progressive course despite initial corticosteroid treatment, the presence of active intraocular inflammation with vitritis and optic disc edema, and the positive response to aggressive immunosuppressive therapy strongly favor RPC over the other conditions [7,8].

Presumed ocular histoplasmosis syndrome (POHS) is not classified within the inflammatory chorioretinopathies, but it should be considered in the differential diagnosis due to the chorioretinal atrophy and pigmentary changes in the posterior pole [9]. The extensive peripapillary atrophy and posterior pole scarring in the left eye are similar to those seen in the end-stage manifestation of POHS, with the geographic distribution localized at the posterior pole aligning with the typical histo spot patterns [10]. However, the presence of active intraocular inflammation - such as vitritis, optic disc edema, and ongoing choroidal inflammation on OCT - undermines POHS as a diagnosis, because these findings are generally inconsistent with POHS, which is characterized by the absence of intraocular inflammation [9,10]. The active, evolving nature of the placoid lesions with serpentine characteristics and the patient's response to immunosuppressive therapy further argue against POHS as a potential diagnosis.

When considering the differential diagnosis for this case, several systemic disease-related chorioretinopathies must be evaluated. SC-like tuberculosis represents a strong differential consideration, given the bilateral posterior pole involvement, choroidal thickening, and serpentine pattern of lesions that closely resemble this patient's presentation [6,11]. The positive Quantiferon test and the chronic, progressive nature with bilateral asymmetric involvement were highly suggestive of a tuberculous etiology; however, further investigations, including chest X-ray and culture, excluded active tuberculosis, making this diagnosis unlikely. Acute syphilitic posterior placoid chorioretinitis may present similarly, with bilateral placoid lesions and optic disc edema [12]; however, this typically occurs in the setting of secondary syphilis with concurrent systemic manifestations, and the absence of systemic or serological evidence of syphilis argues against acute syphilitic disease [6]. Toxoplasmosis retinochoroiditis is another important consideration, particularly due to its tendency to cause retinal lesions with associated vitritis, but the typical presentation includes focal necrotizing retinitis with adjacent chorioretinal scars, vitreous inflammation, and satellite lesions - features that are not consistent with the patient's case description [13]. Ocular sarcoidosis, although it can present with multifocal chorioretinal lesions and optic nerve involvement, is not supported in this case due to the absence of characteristic granulomatous inflammation and choroidal nodules, as well as the lack of systemic findings or supportive laboratory data (e.g., angiotensin-converting enzyme (ACE) levels and chest imaging) [14].

Masquerade syndromes, such as choroidal neoplasm infiltrations, were another important consideration in the differential diagnosis. Although this condition may present with a similar clinical picture - consistent with both the patient’s subjective symptoms and fundoscopic findings - a neoplastic etiology was considered unlikely in this case. This was supported by the patient's relatively young age and the presence of vitritis, which pointed toward an inflammatory origin. Furthermore, a brain magnetic resonance imaging (MRI) was performed to definitively exclude a potential neoplastic process.

## Conclusions

This case study presents an exceptionally rare and diagnostically challenging clinical condition that requires careful differentiation from a wide range of other inflammatory chorioretinal diseases in order to achieve a favorable therapeutic outcome. In this patient, the most critical factors contributing to the correct diagnosis were the characteristic clinical presentation of overlapping lesions, their chronic course, and the lack of clinical improvement following a prior, over-a-month-long corticosteroid monotherapy. It is important to emphasize the value of timely diagnosis and the early initiation of appropriate therapy. In the context of RPC, the mainstay of effective management is triple IMT, consisting of systemic corticosteroids, a calcineurin inhibitor, and a purine antimetabolite. This patient’s history suggests that the first disease episode occurred nine years earlier, but the condition was misdiagnosed and, consequently, inadequately treated at that time. It is likely that earlier recognition and intervention might have preserved better visual function. However, during the present course, disease progression was successfully controlled, and the inflammatory lesions were brought into remission.
